# Unpolarized Release of Vaccinia Virus and HIV Antigen by Colchicine Treatment Enhances Intranasal HIV Antigen Expression and Mucosal Humoral Responses

**DOI:** 10.1371/journal.pone.0024296

**Published:** 2011-09-15

**Authors:** Yan Zhang, Jingyi Yang, Rong Bao, Yaoqing Chen, Dihan Zhou, Benxia He, Maohua Zhong, Yaoming Li, Fang Liu, Qiaoli Li, Yi Yang, Chen Han, Ying Sun, Yuan Cao, Huimin Yan

**Affiliations:** 1 Mucosal Immunity Research Group, the State Key Laboratory of Virology, Wuhan Institute of Virology, Chinese Academy of Sciences, Wuhan, China; 2 The State Key Laboratory of Virology and Modern Virology Research Center, College of Life Sciences, Wuhan University, Wuhan, China; Kyushu Institute of Technology, Japan

## Abstract

The induction of a strong mucosal immune response is essential to building successful HIV vaccines. Highly attenuated recombinant HIV vaccinia virus can be administered mucosally, but even high doses of immunization have been found unable to induce strong mucosal antibody responses. In order to solve this problem, we studied the interactions of recombinant HIV vaccinia virus Tiantan strain (rVTT-gagpol) in mucosal epithelial cells (specifically Caco-2 cell layers) and in BALB/c mice. We evaluated the impact of this virus on HIV antigen delivery and specific immune responses. The results demonstrated that rVTT-gagpol was able to infect Caco-2 cell layers and both the nasal and lung epithelia in BALB/c mice. The progeny viruses and expressed p24 were released mainly from apical surfaces. In BALB/c mice, the infection was limited to the respiratory system and was not observed in the blood. This showed that polarized distribution limited antigen delivery into the whole body and thus limited immune response. To see if this could be improved upon, we stimulated unpolarized budding of the virus and HIV antigens by treating both Caco-2 cells and BALB/c mice with colchicine. We found that, in BALB/c mice, the degree of infection and antigen expression in the epithelia went up. As a result, specific immune responses increased correspondingly. Together, these data suggest that polarized budding limits antigen delivery and immune responses, but unpolarized distribution can increase antigen expression and delivery and thus enhance specific immune responses. This conclusion can be used to optimize mucosal HIV vaccine strategies.

## Introduction

AIDS (acquired immune deficiency syndrome) remains the great unconquered threat to global public health. An effective HIV vaccine is urgently needed. Ninety percent of AIDS cases are transmitted through mucosal routes, such as sexual contact [1]. Targeting immune responses to the site of viral entry can protect the body and help clear the viral reservoir before HIV dissemination [2], [3]. There is evidence that HIV-specific S-IgA could neutralize HIV in the cervicovaginal lavage fluids of uninfected partners of highly exposed persons [4], [5], [6], [7], [8]. The SIV-specific CTLs, which are induced by immunization with an attenuated SHIV, were found to be associated with protection against vaginal challenge in rhesus macaques [9]. Most researchers believe that a vaccine against HIV-1 would need to induce a neutralizing antibody, CD4^+^ Th, and competent CD8^+^ CTL responses at the initial site of viral entry. Systemic vaccination is not sufficient to generate effective compartmentalized mucosal immunity [2], [10].

However, inducing robust mucosal immune responses is difficult. Several factors contribute to successful mucosal immunity [11], [12], [13]. The key factors are as follows: (i) effective delivery of the antigen to the mucosal immune induction site; (ii) enhancement of the mucosal immune responses via safe mucosal adjuvants; (iii) regimen and route of immunization that induce protective responses at the desired mucosal site, preferably systemically; and (iv) adequate vaccine formulation [14].

The vaccinia virus (VV) has been used as a smallpox vaccine. It helped eradicate smallpox worldwide. As a live vaccine vector, it was used for the prevention and eradication of many infectious diseases due to its broad host range, large foreign DNA capacity, lack of carcinogenicity, and ability to induce the expression of foreign antigens in eukaryotic cells [15], [16], [17]. The recombinant wild vaccinia virus is generally avoided for safety reasons, particularly with regard to young children and immune-compromised individuals [18], [19]. As a result, in China, Chen and his colleges developed a series of highly attenuated vaccinia virus Tiantan strains by decreasing the neurotoxicity of the virus [20], [21], [22]. Preliminary experiments demonstrated that the highly attenuated vaccinia virus could induce both humoral and cell-mediated immune responses [20]. One intranasal immunization of this virus protected animals from challenge with the pathogenic vaccinia WR strain [21]. The National Center for AIDS/STD Control and Prevention (China) developed an AIDS vaccine candidate using DNA priming and rVV Tiantan boost with env and gag-pol of CRF07_B'/C as inserts. It has been approved by the SFDA for a phase I trials. Volunteer recruitment started in Beijing in November of 2007 [23], [24].

Unfortunately, highly attenuated recombinant vaccinia viruses can induce only limited mucosal immune responses specific to foreign antigens, even at high doses. Enhancing these immune responses or reducing the necessary dose, would render the vaccine easier to make and safer for patients. Regarding obstacles to mucosal immunization, the first line of opposition that a vaccine encounters is the long-lined mucosal epithelium. There have been some reports that measles and Junin viruses infect the polarized epithelium and are released in a polarized way [25], [26], [27]. We performed this study to evaluate interactions between recombinant vaccinia viruses and epithelial cells and to determine whether epithelial cells released HIV antigens in a polarized way. Upon finding that they did, we performed more tests to see what influence this polarized release has on antigen delivery and specific immune responses. Here we report that recombinant HIV vaccinia virus can infect the mucosal epithelium *in vitro* and in BALB/c mice; both the progeny vaccinia virus and HIV antigen are preferentially released from the apical surfaces and colchicine treatment causes unpolarized antigen expression, delivery, and enhanced specific immune responses. These results provide guidance for immune enhancement and dose reduction in HIV vaccination.

## Materials and Methods

### Cell lines

Caco-2 human colonic carcinoma cell line and Vero cell line were obtained from American Type Culture Collection (Rockville, MD, U.S.). Caco-2 cells were maintained in DMEM (Gibco) supplemented with 10% (v/v) fetal bovine serum (FBS; Gibco), 12.6 mM HEPES, 100 U/ml penicillin, 100 µg/ml streptomycin (Gibco, 1% P+S), and 1×nonessential amino acids (MEM-NEAA, 10 mM, 100X, Gibco). Vero cells were maintained in an otherwise identical DMEM medium containing NEAA. Both were incubated at 37°C in a humidified atmosphere of 5% CO_2_.

### Mice

Female BALB/c mice, 6–8 weeks old, were purchased from the Hunan SJA Laboratory Animal Co., Ltd. and maintained in the Animal Center (Wuhan Institute of Virology, Chinese Academy of Sciences) under specific pathogen-free (SPF) conditions. Female mice were chosen because previous studies have suggested that nasal priming does not work well in male mice [28]. All experiments were approved by the Wuhan Institute of Virology Experimental Animal Ethics and Welfare Committee, the permit number is A2008-019.

### Viral preparation

rVTT-gagpol vaccinia virus was provided by Dr. Zhi-wei Chen (Director of HKU-AIDS Institute) and constructed by Dr. Ke Zhuang (College of Life Sciences, Wuhan University). The virus was homologously recombined with the gagpol gene from HIV-1 B′ and a gfp gene to replace most of the vaccinia virus HA gene. It was propagated in Vero cells. Cells were frozen and thawed three times and centrifuged at 2000 rpm for 20 minutes. Supernatants were aspirated, aliquoted, and stored at −70°C.

Viruses were purified and enriched for immunization. Large-scale propagation of viruses from Vero cells and the supernatant of frozen and thawed cells was added to the 36% (m/v) sucrose solution and centrifuged at 18,000 g for 80 minutes at 4°C. The deposits were suspended, aliquoted at about 1×10^9^ PFU/ml in PBS, and stored at −70°C.

The viral titer was determined by plaque assay of Vero cells.

### Immunization

**Figure 1:**
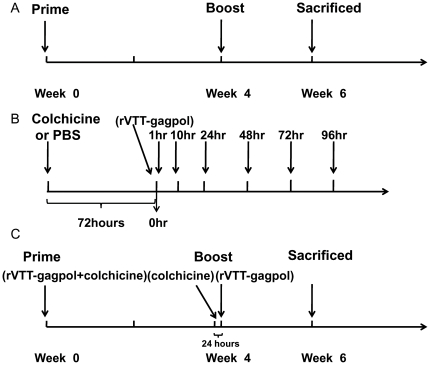
Immunization schemes. (A) I.n. immunization. (B) Colchicine i.n. immunization for distribution of virus and antigen. (C) Colchicine i.n. immunization for antibody tests.

Mice were primed and boosted with 10^7^ PFU/mouse of rVTT-gagpol (12 µl) at 0 and 4 weeks and euthanized at week 6 (Fig. 1A).

For tests of viral and antigen distribution, mice in the colchicine group were i.n. inoculated with colchicine (Duchefa, 20 µg/mouse, 1.25 µg/µl, 16 µl) 72 hours before rVTT-gagpol (10^7^ PFU/mouse, 12 µl) immunization. In the control group, PBS replaced colchicine (Fig. 1B).

Regarding specific antibody response tests, mice in the colchicine group were i.n. primed by rVTT-gagpol (10^7^ PFU/mouse, 12 µl) and colchicine (40 µg/mouse, 10 µg/µl, 4 µl) and then i.n. boosted with colchicine (4 µg/mouse, 1 µg/µl, 4 µl) 24 hours before immunization with rVTT-gagpol (10^7^ PFU/mouse, 12 µl) at week 4. They were euthanized at week 6 (Fig. 1C).

I.n. immunization was performed under anesthesia by 0.06–0.07 mg/g pentobarbital. There were 4–7 mice in each group.

### Collection of serum and mucosal samples

Before euthanasia, vaginal washes were collected. Forty microliters of PBS was aspirated into the vaginal tract using 200-µl tips. The fluid was blown and aspirated 10 times and washed twice. Then serum was obtained. For collection of lung bronchial washes (BW), the trachea was exposed surgically and breast diaphragm was opened for subsequent lung expansion. A very small cut was made on the trachea with small surgical scissors. A 1-ml tip covered with a 10-µl tip from a 1-ml pipette was used to aspirate 500-µl PBS with 1% BSA. The apparatus was carefully inserted into the trachea with the tip toward to lung. This was blown and aspirated three times, slowly, to completely wash the whole lung and bronchia. Both vaginal washes and BW were centrifuged at 2000 rpm for 10 minutes before being stored at −70°C.

### Kinetics of antigen distribution *in vivo*

Mice were immunized and euthanized 1, 12, 24, 48, 72, or 96 hours later. The collecting method was similar to that used previously [29]. Heparinized (20 U/ml) blood was collected and centrifuged at 2000 rpm. Nasal washes and lung bronchial washes were collected in 1 ml DMEM with 1% (P+S) and 10% FBS. The method of collecting bronchial washes is described as above. For nasal washes, the initial steps were the same as those used for bronchial washes, but after the small cut was made on the trachea, a 1-ml tip covered with a 10-µl tip from a 1-ml pipette was carefully inserted into trachea with the tip toward the nostril, and solution was to made to flow out of nostrils. It was washed twice. The nasal turbinate and lungs were separately homogenized in 1 ml DMEM with 1% (P+S) and 10% FBS. Washes and homogenized samples were centrifuged at 2000 rpm for 10 minutes. The vaccinia viral titer and p24 concentration were determined by plaque assay and ELISA, respectively.

### Immunohistochemical staining of p24 in mice

After immunization, mice were bled and euthanized. The nasal turbinate and lungs were collected and fixed in 10% (v/v) formaldehyde for 6–12 hours. The decalcification of nasal turbinate was performed in cooled 5% (v/v) nitric acid in a microwave oven [30]. Decalcification solution was maintained at 4–10°C by immersing it in a beaker with ice, which was refreshed every 5–10 minutes for 1 hour. Then both the lung and the decalcified nasal turbinate samples underwent serial dehydration and were imbedded in paraffin and cut into slices 2 µm thick.

The tissue sections were heated, rehydrated, and subjected to antigen retrieval procedures, including microwaving in 0.01 M citrate buffer (pH 6.0) and incubation in 3% hydrogen peroxide in deionized water. The following experiment was performed using ZSGB-BIO, sp-9001 Histostrain™-Plus Kits: After tissue sections were blocked for 30 minutes at room temperature, they were incubated with rabbit anti-p24 (1∶3000, gift of Dr. Bing Yan, Wuhan Institute of Virology, Chinese Academy of Sciences) for 2 hours at 37°C. Then the tissue sections were incubated with biotin-labeled goat anti-rabbit antibody and strep-HRP separately for 15 minutes at room temperature. They were then visualized using diaminobenzidine (DAB) kit (Maixin-Bio). Before primary antibody incubation, the samples were washed three times with PBS. After primary antibody incubation, they were washed three times with PBS with 0.02% (v/v) Tween 20 and twice with PBS alone. All slides were counterstained with hematoxylin solution. Slides were photographed in an OLYMPUS BX51 microscope equipped with QImaging CCD camera (MiroPublisher 5.0 RTV) and QCapture vision software.

### Plaque assay

The experiment was performed under a biosafety hood using aseptic technique. Vero cells were seeded in 24-well plates and incubated for 24 hours. Serial 10-fold dilutions were inoculated for 2 hours. Inocula were removed and washed with 0.5 ml PBS. Then freshly prepared 1% (m/v) agarose and 2×MEM (Gibco, 6% FBS+1% (P+S)) were added. Each plate was incubated for 48 hours, and 0.2 ml of 5 mg/ml crystal violet in 10% formaldehyde was added to fix and stain the cell layers for 2 hours at room temperature. Then the agarose was carefully flushed out with tap water and the plates were air-dried. Plaques were counted. PFU (plaque formation unit) = dilution×P× (1/V), (P: plaque counts; V: volume of inocula).

### ELISA (anti-p24 antibody and p24 antigen detection)

For anti-p24 antibody detection, 5 µg/ml *E. coli* recombinant p24 protein was coated in 0.1 M carbonate bicarbonate buffer (pH 9.6) at 37°C for 2.5 hours. Then plates were blocked with 1% BSA in PBS at 4°C overnight. Serial 4-fold dilutions of sample in PBS (0.5% BSA) were added to the plates and incubated for 2 hours at 37°C. Then goat anti-mouse IgG-AP (1∶2000, Southern Biotech) or goat anti-mouse IgA-AP (1∶2000, Southern Biotech ) in PBS (0.5% BSA) was added and incubated for 1 hour at 37°C. The colorimetric conversion was performed using Sigma Fast™, p-nitrophenylphosphate (PNPP) tablet sets (Sigma, N-2770) for 1 hour at 37°C. After each incubation (but not after blocking), plates were washed with 0.05% Tween 20 in PBS. Plates were evaluated in a microplate spectrophotometer (Multiskan MK3, Thermo LabSystems) at 405 nm and the readouts were calibrated with blanks. Titers of the samples were calculated from endpoint dilutions twice the negative value.

For p24 protein detection, the double antibody sandwich method was used. Diluted rabbit anti-p24 poly-antibody (1∶500) was coated in 0.1 M carbonate bicarbonate buffer (pH 9.6) at 37°C for 2.5 hours. Then plates were blocked using 10% FBS in PBS at 4°C overnight. Plates containing treated samples were serially 2-fold or 4-fold diluted with PBS containing 10% FBS and 1% (v/v) TritonX-100 and treated for 3 hours at 37°C to release p24 from the HIV-virus-like particle. *E. coli* recombinant p24 diluted in the same manner was used to create a standard curve to determine the concentration of p24 in samples. Then treated and untreated sample dilutions were placed in wells for 2 hours at 37°C. Mouse anti-p24-HRP antibody (1∶1200) was added and incubated for 1 hour at 37°C. The colorimetric conversion of TMB substrate reagent was used for 10–20 minutes at room temperature. The working solutions were as follows: 250 µl solution B +10 ml buffer A +3.75 µl 30% H_2_O_2_. Buffer A: 205 mM potassium citrate (pH 4.0). Solution B: 41 mM TMB in DMSO [31]. After each incubation, but not after blocking, plates were washed with 0.05% Tween 20 in PBS. Colorimetric conversions of the substrate were read at 450 nm and 570 nm. OD value  =  (OD value at 450 nm) − (OD value at 570 nm). The p24 standard curve was drawn and a linear regression equation was made. The linear range was 0.005 ng/ml to 0.1 ng/ml. The p24 concentration of the sample was computed using the equation.

### Polarized Caco-2 monolayers

Monolayers were grown in 12-well Corning Costar Transwell plates (Corning Inc., U.S.) with 0.4-µm or 3-µm pore filter inserts. Cells were cultured for 14–16 days after becoming confluent (which took about 2 days). The medium was changed every other day so that would be well polarized. The TER readings were about 600 Ohm/cm^2^ (as measured by a Millicell-ERS voltmeter; Millipore Corp., U.S.).

### rVTT-gagpol infection of Caco-2 cells

Polarized Caco-2 monolayer cells were infected with rVTT-gagpol apically at MOI (multiplicity of infection) 1. Virus was added directly onto the apical surface and incubated at 37°C in 3% FBS-containing DMEM medium. The same medium was added to the basolateral compartment. Virus was aspirated after 2 hours. The apical surface was washed three times with PBS. Then the cultures were fed fresh medium and returned to the incubator. Infected polarized Caco-2 monolayer cells were sampled at 0, 24, 48, and 72 hours post-infection. Apical, basolateral media were collected. Cells were frozen and thawed three times. Finally, three types of samples from apical media, basolateral media, and cells were aliquoted and stored at −70°C.

In the unpolarized group, starter cells were seeded in transwells for 3 days and then infected with rVTT-gagpol. In the colchicine-treated group, colchicine (0.08 mg/ml) was added to polarized Caco-2 cell layers 72 hours before infection. Forty-eight hours after infection, apical and basolateral media were collected and fresh medium was added. All samples were collected at 72 hours. The p24 and viral titer were determined by ELISA and plaque assay, respectively. The virus titer this time was calculated as the sum of that collected at 48 hours and at 72 hours. Experiments were performed double or in triplicate.

### Electron microscopy

Each monolayer was rinsed with PBS and fixed with 2% formaldehyde and 0.1% glutaraldehyde in 0.1 M sodium phosphate buffer, pH 7.2, at 4°C for 2–4 hours [32]. Following fixation, the cells on filters were cut free of the plastic inserts, postfixed in OsO_4_, contrasted en bloc with 1% uranyl acetate, dehydrated in a graded series of ethanol solutions, and embedded in epon. Sections were cut perpendicularly to the filter, collected on Formvar-coated mesh grids, and examined under a JEM-1230 electron microscope.

### Statistical analyses

All statistical analyses were conducted using GraphPad Prism, version 4. Statistical comparisons were conducted using the 2-tailed unpaired t test. P values <0.05 were considered significant.

## Results

### Effects of high doses of rVTT-gagpol on levels of mucosal humoral immune response after intra-nasal immunization

**Figure 2:**
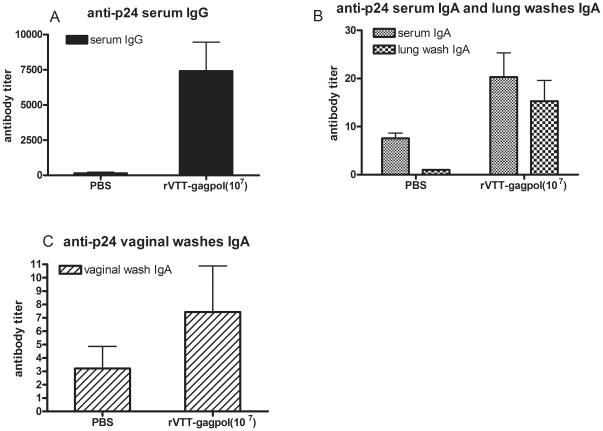
Humoral immune responses to high doses of rVTT-gagpol after i.n. inoculation. Mice were i.n. inoculated with rVTT-gagpol at weeks 0 and 4 and euthanized during week 6. Sera, lung washes, and vaginal washes taken during week 6 were collected to ascertain anti-p24 IgG and IgA by ELISA. There were seven mice in each group. Anti-p24 (A) serum IgG, (B) serum IgA, lung wash IgA, and (C) vaginal wash IgA are displayed above. Three independent experiments were conducted with similar results. The results of one representative experiment are shown.

To evaluate the mucosal immune responses associated with rVTT-gagpol, BALB/c mice were intra-nasally administered 10^7^ PFU of rVTT-gagpol (Fig. 2). During week 6, after two immunizations four weeks apart, mice were euthanized and antibody levels were determined. In detail, the anti-p24 serum IgG titer was about 7500, the serum IgA titer was about 20, the lung wash IgA titer was about 18, the vaginal wash IgA titer was about 7, and no significant differences from the PBS group were found (Fig. 2). The anti-p24 IgG titer in lung washes was found to be higher than that of IgA (data not shown). However, IgG levels were not stable at the mucosal surface [33]. Vaginal washes were not evaluated for IgG. This showed that high-dose rVTT-gagpol immunization could induce high levels of p24-specific IgG in serum but only low IgA titers, suggesting a weak mucosal immune response.

These results were similar to those reported by other groups: In one study, 5×10^6^ PFU/mouse of recombinant vaccinia virus was given to mice at 0 and 4 weeks, and these mice were then euthanized and sampled at 6 weeks [34]. The specific IgA in lung washes was shown via OD values and could be converted to about 20 titer. Another group gave its mice 2×10^6^ PFU i.n. only once and euthanized them 3 weeks later. Their specific antibody results were as follows: serum IgG 2 100±1,370, serum IgA 8.0±4.2, bronchial wash IgA 24.0±26.3 [29].

Although rVTT-gagpol is a replicating virus vaccine, it still needs high doses to induce even small mucosal immune responses after mucosal immunization. In order to find the reasons behind this and ways to enhance immune response and reduce the dose, we studied the interactions between rVTT-gagpol and polarized epithelia.

### Release of rVTT-gagpol and HIV antigen p24 from epithelial cells *in vitro*

It is known that epithelial cell layers are polarized *in vivo*. *In vitro*, mucosal epithelial cell lines cultured on a permeable membrane called a transwell can become polarized monolayers. In this study, the human colonic carcinoma Caco-2 cell line was cultured and developed into polarized cell layers. rVTT-gagpol was inoculated from the apical side. Then small, local cytopathic effects (CPEs) appeared in the cell layer: Some loose connections were formed between neighboring cells but these had no great effect on the integrity of the monolayer. The infected cell monolayer was fixed and processed for electronic microscopy. p24 concentrations and rVTT viral titers in the apical, basolateral, and cell samples were determined at various time points.

**Figure 3:**
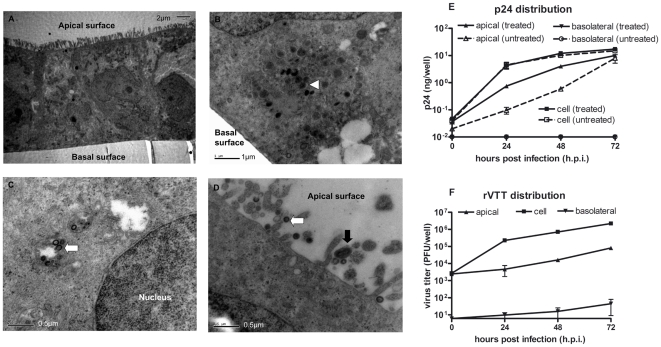
Infection, expression, and distribution of rVTT-gagpol in polarized Caco-2 monolayer *in vitro.* Sixteen days after polarization, Caco-2 cell monolayers were infected by rVTT-gagpol at MOI 1 from the apical surface. At various times, monolayers were fixed and prepared for electronic microscopy observation, as shown in images (A), (B), (C), and (D). For infection and distribution kinetics, the p24 content and virus titers of the samples were determined by ELISA and plaque assay, respectively, as shown in images (E) and (F). In detail: (A) Caco-2 monolayer on filter at 48 hours post infection (scale bar: 2 µm). (B) Immature vaccinia virus (**▹**) at 72 hours post infection (scale bar: 1 µm). (C) HIV VLP (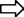) about100 nm in diameter at 72 hours post infection (scale bar: 0.5 µm). (D) HIV VLP (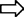) and mature vaccinia virus (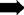) about 200 nm in short diameter and about 400 nm in long diameter released from apical surface (scale bar: 0.5 µm). (E) p24 distribution. Treated samples were treated with 1% TritonX-100 before testing to release the internal p24 of VLP. (F) rVTT virus distribution. Kinetics experiments were performed in triplicate, three for each noted point in time. Each electron microscopy observation was performed twice.

The polarized cell monolayer was observed under electronic microscopy (Fig. 3A). Some viral factories were present in the cell plasma, in which there were many round immature vaccinia viruses of about 200 nm in diameter (Fig. 3B). Some of the gagpol assembled HIV-virus-like particles (VLP), about 100 nm in diameter, were in the process of being transported (Fig. 3C) and some were outside of the cells' apical surface (Fig. 3D). Mature vaccinia viruses, about 200 nm in short diameter and 400 nm in long diameter, were observed both inside the cells (not shown) and outside of the apical surface (Fig. 3D). No vaccinia viruses were seen outside the basolateral surface.

The kinetics of p24 and virus distribution post-infection are shown in Figures 3E and F. The concentrations of dissociative p24 and p24 in VLP were determined using double antibody sandwich ELISA. Disassociated p24 was found to be expressed in the cells and released from the apical but not the basolateral surface. The p24 in VLP was evaluated using 1% TritonX-100. Results suggested that increased levels of cellular and apical p24 were released from the apical surface as well. Basolateral VLP p24 still could not be detected. For total p24, about 10 ng/well was found to be present in the cells, about 1–8 ng/well was present in the apical medium, and none was detected in the basolateral medium.

Viral titer was determined by plaque assay. The virus was found to replicate at about 10^6^ PFU/well in cells and was released from the apical surface at about 10^4^–10^5^PFU/well. This is about 1000 times the level in the basolateral medium. Even when the medium was changed every 48 hours, the kinetics showed similar trends (data not shown).

After rVTT-gagpol infection, polarized Caco-2 cell layers assembled progeny vaccinia viruses, expressed HIV genes, and released viral products from the apical surface. Whether the situation would be the same *in vivo* and what impact this preferential release might have on specific immune responses remained to be determined. To this end, we studied the interactions between rVTT-gagpol and the epithelium in BALB/c mice.

### Locations of rVTT-gagpol replication and HIV antigen expression in nasally immunized mice

To identify the amount and location of HIV antigen, mice were i.n. immunized with rVTT-gagpol and euthanized 1, 10, 24, 48, 72, or 96 hours later. Then plasma, nasal washes, lung bronchial washes, and homogenized nose and lung tissue samples were collected. Levels of virus and p24 in these samples were detected by plaque assay and ELISA, respectively.

**Figure 4:**
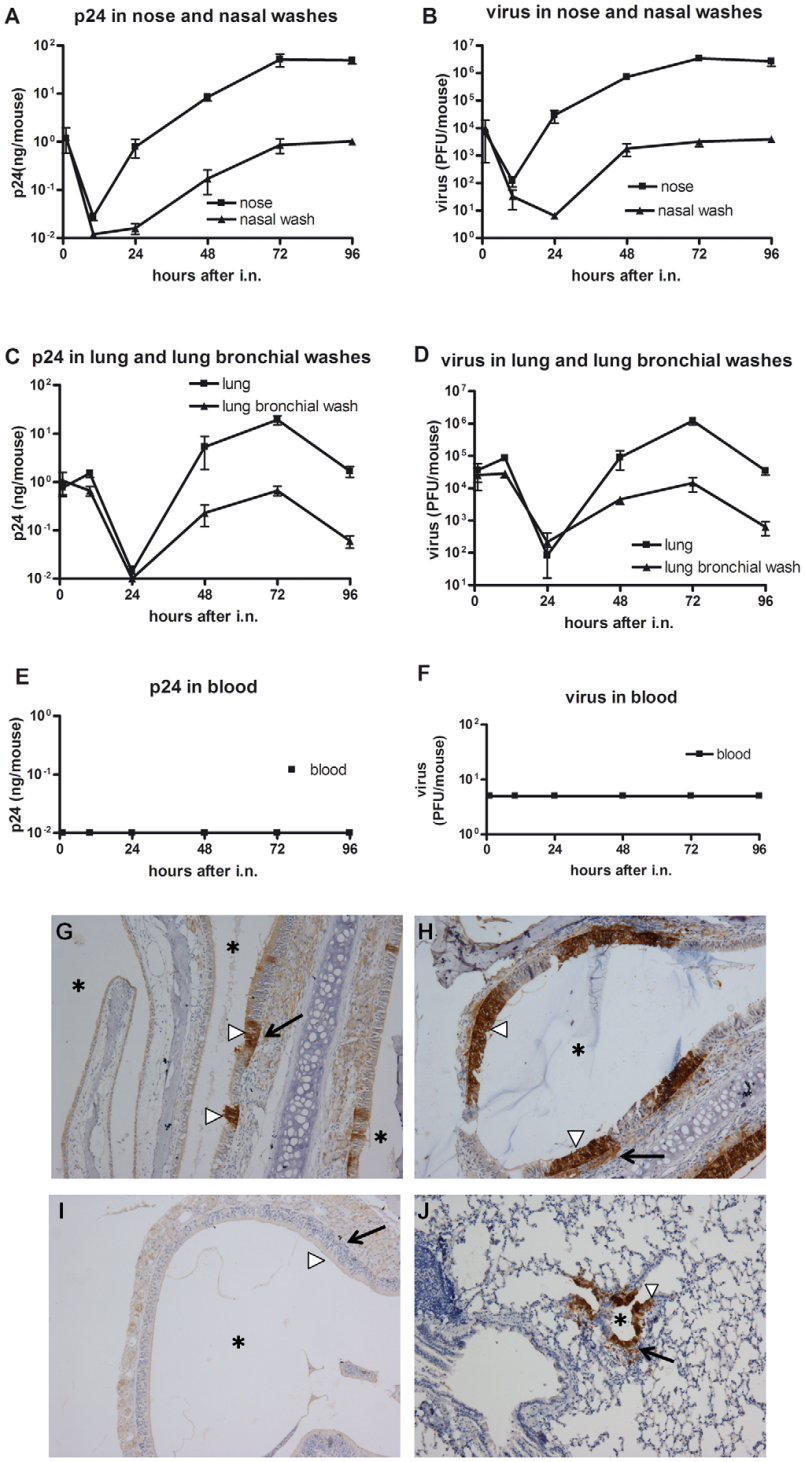
Distribution of p24 antigen and vaccinia virus in mice. Six-week-old female BALB/c mice were inoculated i.n. with 1×10^7^ PFU of rVTT-gagpol per mouse. For determination of infection and expression kinetics, nose samples, nasal washes, lung samples, lung bronchial washes, and plasma were collected at specific times. p24 and virus titer were determined by ELISA and plaque assay, respectively (A, B, C, D, E, and F). For immunoperoxidase staining of p24 antigen, nose and lung tissues were fixed and prepared into paraffin slides, which were stained with immunoperoxidase (G, H, I, and J). Rabbit anti-p24 served as the first antibody. In detail, p24 distribution in (A) nose samples and nasal washes, (C) lung samples and lung bronchial washes, and (E) plasma. Virus distribution in (B) nose samples and nasal washes, (D) lung samples and lung bronchial washes, and (F) plasma. Nose section (G) 24 hours and (H) 72 hours after i.n. inoculation with rVTT-gagpol. (I) Nose section after PBS i.n. inoculation. (J) Lung section 72 hours after i.n. inoculation. Magnification, 100×. (**▹**) designates epithelium, (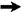) designates lamina propria, (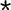) and designates lumen. These images are typical of those observed. There were five mice in each group. The experiments were performed in triplicate.

Results showed that after mice were i.n. inoculated, the virus replicated and p24 was expressed in respiratory system but not in the blood (Figs. 4A, B, C, D, E, and F). Viruses entered the nose tissue. Viral levels decreased to their lowest point (10^2^ PFU) at about 10 hours and then increased through the first and second days, reaching a peak at 72 hours (about 5×10^6^ PFU). At the same time, viruses were released into the lumen of the nose, where viral levels reached their lowest point (about 8 PFU) at 24 hours and their highest point (about 10^3^ PFU) at 72 hours (Fig. 4B). p24 expression and distribution took on similar trends. The largest amount of expression was achieved, about 70 ng per mouse, at 72 hours (Fig. 4A).

Viruses were able to reach the lungs when the volume of i.n. administration exceeded 10 ul. Then the viruses replicated and p24 was expressed in the lungs (Figs. 4D and C). The lowest virus and p24 levels were detected at 24 hours and the highest at 72 hours. Viral titers were found to reach 10^6^ PFU, lower than that in nose tissue. For p24, the amount reached 30 ng. If the volume of i.n. administration was 8 ul, fewer viruses reached the lungs and peak viral titer only reached 10^5^ PFU per mouse (data not shown). However, neither virus nor p24 was detected in the blood (Figs. 4E and F). This shows that infection and expression were restricted to the respiratory system.

In order to determine the exact location of p24 antigen in the nose and lungs, we performed immunoperoxidase staining of p24 antigen on mice inoculated with rVTT-gagpol (Figs. 4G, H, I, and J). At 24 hours, there was a little p24 in the epithelium of nose (Fig. 4G). At 72 hours, there was more p24 but large amounts remained in the epithelium and only a small amount was found in lamina propria (Fig. 4H). In the lungs, little p24 was observed at 24 hours (image not shown). A certain amount of p24 was observed in the epithelia of the lungs at 72 hours and some was delivered to the alveolar cells (Fig. 4J). The p24 immunohistochemistry results were considered representative of viral distribution.

rVTT-gagpol was found capable of infecting the epithelial cells of the nose and lungs. Progeny virus and p24 were constantly released into the lumen, though only a little reached the lamina propria. These results correspond with those of our *in vitro* experiments. Viral infection and expression were detected in the respiratory system but not in the blood. As far as a vaccine development is concerned, it was expected that antigen would be delivered primarily into the whole body but not outside it. The limited efficiency of antigen delivery was found to be due to polarized distribution in the epithelium. This also suggests that the polarized release is a barrier to the induction of an efficient immune response. One approach to improve the situation would be to find a way to induce unpolarized release of the virus and HIV antigen.

### Effects of colchicine on unpolarized distribution of rVTT and HIV antigen *in vitro* and in mice

**Figure 5:**
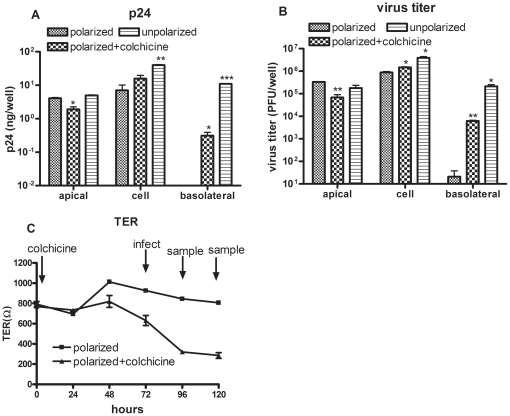
Comparison of distribution of differently treated cell layers after rVTT-gagpol infection *in vitro*. Caco-2 cell layers were infected with rVTT-gagpol 14–16 days after polarization to form the polarized group. A similar group was treated with colchicine (0.08 mg/ml) apically 72 hours before rVTT-gagpol infection to form the polarized + colchicine group. A 3-day Caco-2 transwell culture was infected with rVTT-gagpol to form the unpolarized group. The data shown are the sums of the 48-hour and 72-hour values. (A) p24 distribution. (B) Virus distribution. (C) TER. Apical, cellular, and basolateral samples of the three groups were compared. T-testing was conducted to compare the polarized + colchicine group and unpolarized group to the polarized group. Statistical significance: (*P*<0.05) in an unpaired test is indicated by an asterisk. There were two wells for every group. The experiments were performed twice.

Polarized budding relies upon the cytoskeleton [26], [35]. When its microtubules are depolymerized, proteins that ordinarily target the apical surface can end up elsewhere [36]. Colchicine, a drug that inhibits microtubule polymerization, can cause unpolarized release of the Junin virus in polarized Vero cells [26]. We used colchicine to inhibit polarized release as follows: After Caco-2 cells were allowed to polarize for 14–16 days, colchicine was added apically 72 hours before rVTT-gagpol infection. This group is here called the polarized + colchicine group. Another polarized group was infected with rVTT-gagpol only. The unpolarized groups consisted of infected, unpolarized cells. Caco-2 cells were cultured in transwell for 3 days, and rVTT-gagpol was then inoculated. The data presented in Figure 5 were calculated as the sum of that detected at 48 hours and 72 hours.

Compared to the polarized-only group, the unpolarized and polarized + colchicine groups showed relatively unpolarized distributions of p24 and virus (Fig. 5). In the unpolarized group, the total amount of virus was significantly higher than in the polarized group. Progeny viruses were released from both the apical and basolateral surfaces (Fig. 5B). In the colchicine-treated group, polarized budding was disturbed. The trend of distribution was similar to that of the unpolarized group. Compared to the polarized group, the amount of virus in the cells was higher, the amount in the basolateral medium was 20 times higher, and the amount in the apical medium was lower (Fig. 5B). p24 distribution showed a similar trend. In the colchicine-treated group, TER decreased in the 3 µm transwell (Fig. 5C) but increased in the 0.4 µm transwell at low concentrations (0.08, 0.4 mg/ml). It decreased at high concentrations (2 mg/ml) (data not shown). In both the 3 µm (Figs. 5A, B) and 0.4 µm transwells (data not shown), colchicine treatment induced basolateral viral and p24 release. In conclusion, colchicine treatment 72 hours before rVTT-gagpol infection was found to cause unpolarized release of virus and p24 *in vitro*.

**Figure 6:**
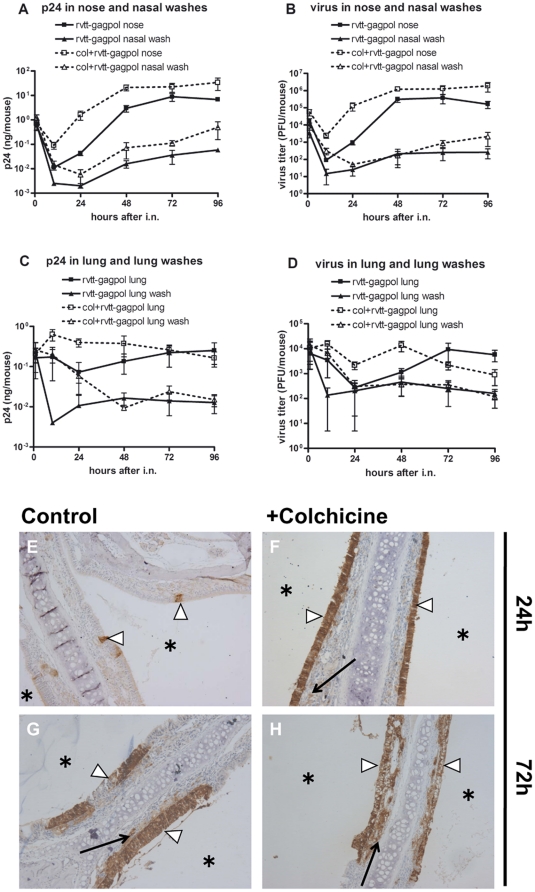
Comparison of p24 and virus distribution between untreated and colchicine-treated rVTT-gagpol immunization groups in mice. The colchicine-treated group included 6-week-old female BALB/c mice that were i.n. inoculated with colchicine (20 µg) 72 hours before rVTT-gagpol immunization (1×10^7^ PFU). The rVTT-gagpol immunization group was given identical treatment except that PBS was used as a substitute for colchicine. Infection and expression kinetics in nose samples, nasal washes, lung samples, and lung bronchial washes were evaluated (A, B, C, and D). Immunoperoxidase staining of p24 antigen in the nose (E, F, G, and H). In detail, p24 distribution in (A) nose samples and nasal washes and (C) lung samples and lung bronchial washes. Virus distribution in (B) nose samples and nasal washes and (D) lung samples and lung bronchial washes. Nose sections of the rVTT-gagpol immunization group after (E) 24 hours and (G) 72 hours. Nose sections of the colchicine-treated rVTT-gagpol immunization group after (F) 24 hours and (H) 72 hours. (▹) designates epithelium, (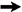) designates lamina propria, and (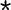) designates lumen. There were five mice in each group and the images shown are typical of those observed. Magnification, 100×. Two independent experiments were conducted with similar results. One set of results is shown.

Next, the impact of colchicine on antigen distribution was explored in mice epithelia. Colchicine was i.n. inoculated 72 hours before i.n. administration of rVTT-gagpol. PBS was used as a substitute for colchicine in the control group. Mice were euthanized at 1, 10, 24, 48, 72, and 96 hours. Then plasma, nasal washes, lung bronchial washes, and homogenized nose and lung tissues were collected. Levels of virus and p24 in these samples were measured by plaque assay and ELISA, respectively. The results demonstrated that, relative to the rVTT-gagpol group, the colchicine-treated group showed consistently higher p24 expression and infection rates in both nose and nasal washes (Figs. 6A and B), about 70 times higher at 24 hours and about 5 times higher at 72 hours. In the lung and lung bronchial washes, both p24 and virus levels were much higher after 48 hours. After 72 hours, both began to decrease, reaching their lowest points at 96 hours (Figs. 6C and D). No virus or p24 was detected in the blood of colchicine-treated or control mice (data not shown).

Regarding p24 distribution in the nose, immunoperoxidase staining of p24 antigen showed that, at 24 hours, the colchicine-treated group expressed p24 in the epithelium at a much higher rate than the group immunized with rVTT-gagpol alone did. The level of p24 in the lamina propria of both groups was low (Figs. 6E and F). At 72 hours, the colchicine-treated group expressed p24 at much higher levels than the rVTT-gagpol immunization group in both the epithelia and the lamina propria (Figs. 6G and H).

To ascertain the toxicity and reversibility of the effects of colchicine, we measured body weight and observed the condition of mice every other day after immunization. Body weight decreased after immunization but was restored about 10 days later (data not shown). The general condition of mice, as evaluated through coat smoothness and activity level, corresponded with changes in body weight.

In conclusion, colchicine treatment was found to depolarize the release of vaccinia virus and p24 both *in vitro* and in immunized mice. In mice, it also increased the level of both. Our next task was to examine specific immune responses.

### Effects of unpolarized release of p24 and rVTT-gagpol in the nasal epithelium on mucosal and systematic immune responses

We established a colchicine treatment group to determine the influence of colchicine treatment on specific immune responses. Mice were i.n. administered rVTT-gagpol (10^7^ PFU) and colchicine (40 µg) at the same time. During the week 4, the mice were inoculated with colchicine (4 µg) 24 hours before rVTT-gagpol inoculation. PBS was used as a substitute for colchicine in the control group. During week 6, mice were euthanized and blood and mucosal samples were collected.

**Figure 7:**
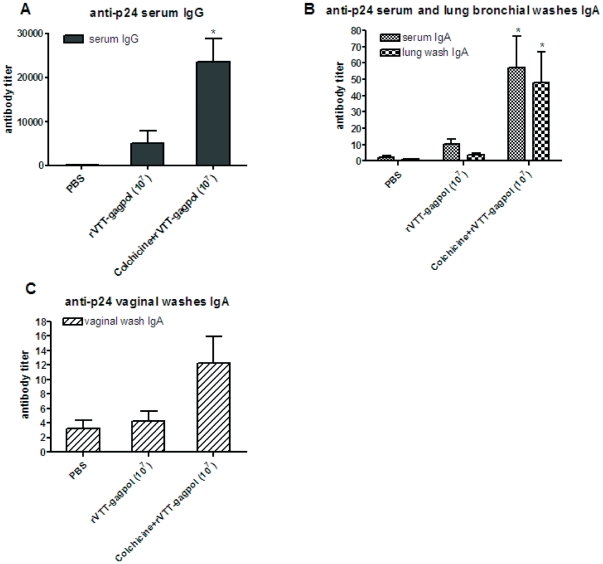
Comparison of anti-p24 humoral immune responses between untreated and colchicine-treated rVTT-gagpol immunization groups in mice. The colchicine-treated group consisted of five 6-week-old female BALB/c mice that were given i.n. rVTT-gagpol (10^7^PFU) and colchicine (40 µg) at the same time. During week 4, mice were given colchicine boosters (4 µg) 24 hours before rVTT-gagpol. The mice were euthanized during week 6. The rVTT-gagpol immunization group was given identical treatment except that PBS was used as a substitute for colchicine. Serum and mucosal samples were collected to test for the presence of anti-p24 antibody by ELISA. Anti-p24 levels of (A) serum IgG, (B) serum IgA, bronchial wash IgA, and (C) vaginal wash IgA are displayed. T-testing was conducted between the colchicine-treated group and the rVTT-gagpol immunization group. Statistical significance (P<0.05 in an unpaired test) is indicated by an asterisk. Two independent experiments were conducted with similar results. One set of results is shown.

In the colchicine group, anti-p24 immune responses were significantly stronger than in the control group (Fig. 7). The serum IgG titer was about 5 times that of the controls. The serum IgA was about 5 times higher. The lung bronchial wash IgA titer was about 10 times higher. The vaginal wash IgA titer was about 3 times higher. Generally, the antibody titers increased depending on the location of induction. The mucosal level of IgA increased and systematic effects on IgA and IgG levels were observed.

Colchicine (20 µg/mouse and 4 µg/mouse) was given to mice to determine its effects on immune responses. We found that the influence on antibody response at 20 µg was similar to that at 40 µg, but 4-µg doses had no significant effects on anything but serum IgA titer, which showed a lower level of increase than that seen at higher doses (data not shown). The conditions of colchicine treatment for detection of antigen distribution differed slightly from those used for evaluation of immune responses. Although we used the same treatment procedure and noted enlarged corresponding immune responses, the results were not stably reproducible. The results shown in Figure 7, however, were both reproducible and consistent with the idea that colchicine enhances immune response. There are many factors that might have influenced the immune responses at some point between antigen distribution and immune response.

These data suggest that the unpolarized release of vaccinia virus and antigens renders p24-specific antibody responses more intense.

## Discussion

The purpose of the present study was to evaluate the interactions between HIV recombinant vaccinia virus (rVTT-gagpol) and the mucosal epithelium and the relationship between antigen distribution and specific immune responses. Even high doses of most vaccinia virus vaccine candidates cannot induce strong mucosal IgA responses, which are related to protection from viral challenge [29]. Other studies have been performed with doses of rVTT ranging from 2×10^6^ to 5×10^6^ PFU/mouse [29], [34], [37]. Only low specific antibody titers were produced [29], [34]. Large doses are harder to prepare and can cause side effects. However, i.n. administration of recombinant vaccinia virus alongside HN or F was found to protect mice from challenge by Sendai virus via the respiratory duct [29]. When inoculated via either intranasal or intraoral routes, recombinant vaccinia virus with S protein from SARS-CoV induced 20–100 times the level of Nab in serum than that induced by rMVA [22]. A means to increase the efficiency and to reduce dose size would be useful to researchers, doctors, and patients interested in this vaccine.

*In vitro* experiments in which rVTT immunization was performed via the mucosa, demonstrated that rVTT-gagpol could infect polarized Caco-2 epithelial layers and that progeny virus and expressed p24 were released mainly from the apical surface (Fig. 3). Vermeer *et al.* also reported that the vaccinia virus infected differentiated human airway epithelia and was released mainly from the apical surface *in vitro* [38]. Huang *et al*. reported that SIV budded basolaterally in polarized Vero C1008 cells [39]. Randall *et al*. reported that, when polarized Vero-pFN cells were infected with VVgag, similar amounts of p24 capsid protein were released into virus particles at the apical and at the basolateral surface. In contrast, when cells are infected with both VVgag and VVenv, 94% of p24 and all gpl20 are associated with particles released into the basolateral medium [40]. These results indicate that the HIV-1 envelope glycoprotein renders virus particles containing the gag protein to be released from the basolateral surface [40]. However, after we co-infected polarized Caco-2 cells with rVTT-gagpol and rVTT-env via the apical surface, no increased associative p24 or VLP p24 was detected in the basolateral medium. We also infected polarized Vero C1008 cells with rVTT-gagpol via the apical surface. The integrity of the cell layers deteriorated 4–12 hours after infection depending on the MOI (data not shown). Before the cell layers broke down, p24 and vaccinia virus were mainly released from the apical surface. After that, more p24 and virus was present in the basolateral medium than in the apical. In contrast, Caco-2 cell layers maintained their integrity after infection: p24 and virus were still released mainly from the apical surface. The reason for this might be that Caco-2 cells are from the epithelial cells of the colon, which is mucosal tissue, while Vero cells are derived from the epithelial cells of kidney. This makes Caco-2 cells more representative of mucosal epithelia *in vivo* than Vero cells.

We verified the interaction in immunized mice. rVTT-gagpol was able to infect respiratory systems, though no viruses or p24 could be detected in the blood (Fig. 4). When mice were immunized with rVTT-gagpol via the vagina, the virus and p24 were restricted to the vaginal system and could not be detected in blood (unpublished data). Other groups found similar results. Recombinant vaccinia virus has been shown to replicate in the nose and lungs after i.n. inoculation without any virus detectable in the blood. However, after i.p. inoculation, some virus was detected in the blood on days 1–4 [29]. Another study focused on the activity of luciferase in various mouse tissues after immunization with recombinant MVA through intranasal (i.n.), intravaginal (i.v.), and intrarectal (i.r.) routes. After i.n. immunization, luciferase activity was detected only in the lungs, NALT, and lung-draining lymph nodes but not in other tissues. In an i.r. immunization model, activity was not detected in the rectum, rectal lymph node, or other tissues. Similarly, after i.v. immunization, no activity was detected in the ovary or vaginal lymph node. Activity in the vagina lumen was not tested [41].

In contrast to the vaccinia virus, the vesicular stomatitis virus (VSV) has been found to be released from the basolateral surface after MDCK infection *in vitro* [42]. After VSV type New Jersey (VSV-NJ) was i.n. inoculated in deer mice, infection of the olfactory epithelium took place on the day 1 and a great deal more virus was observed in the lamina propria on the day 4. Of the juvenile and adult deer mice studied, 49% developed virusemia on days 1, 2, or 3 [43]. These results imply that VSV infects the epithelium first and then spreads to the lamina propria and blood. Unlike VSV, vaccinia viruses are mainly released to the lumen, and it is difficult for them to enter the blood through the lamina propria.

Because the polarized release of rVTT-gagpol and p24 antigen limits antigen delivery and specific immune responses, we used colchicine to increase its effectiveness. We showed that colchicine acts by disrupting polarized release *in vitro* (Caco-2 cell layers) and in immunized mice (Figs. 5 and 6). *In vitro*, the 3-µm pore in the transwell membrane was used to make sure that rVTT was released only from the basolateral surface. The 0.4-µm transwell pore was able to block basolateral release, showing only 1/10^3^ as many basolaterally released viruses as samples equipped with 3-µm pores. The release of p24 antigen was not significantly different (data not shown). This implies that, due to the large size of the vaccinia virus, it passed through 3-µm transwell pores more easily than through 0.4-µm pores.

**Figure 8:**
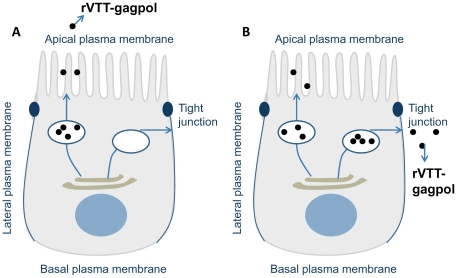
rVTT-gagpol released from (A) the apical surface and (B) the basolateral surface (referenced and modified from [**35**]).

In mice, colchicine treatment increased viral infection and expression of p24 in the nose, nasal washes, and lamina propria (Fig. 6) with some damage to the epithelium. This is consistent with the situation *in vitro*, in which cell layers turned out some plaques but maintained the integrity of the epithelial layer relatively well. One explanation of how colchicine treatment was able to increase the rates of infection and expression may be this: Colchicine influenced dissemination between cells during the second period of infection. During rVTT-gagpol infection of the epithelial cells, progeny viruses were released from the apical surface, from which they infected neighboring cells, again via the apical surface (Fig. 8A). Because transportation relies on microtubules, depolymerization of those microtubules by colchicinie caused some vesicles to target the basolateral surface (Fig. 8B) [36]. The basolaterally targeted vesicles fused with the upper two thirds of the lateral surface, not just the region immediately beneath the tight junction [36]. In addition, vaccinia virus more readily infects differentiated human airway epithelia from the basolateral surface than from the apical surface [38]. In this way, colchicine treatment rendered infected cells much more likely to infect other cells.

Our results demonstrated that the unpolarized release of virus and antigen increased specific antibody responses. Other studies have described the impact of colchicine on immune responses. Colchicine can act as an immune suppressor at high doses (100 µg/ml) and an immune enhancer at low doses (0.5–1 µg/ml) [44]. It can increase immune responses when co-administered with antigens, enhancing immune response by about a factor of two [44]. In this study, the doses were 80 µg/ml *in vitro* and 4–40 µg/mouse (1000–10,000 µg/ml). Previous reports indicate that both would result in immune suppression. We found that colchicine does not act as an immune enhancer under these conditions. In addition, immune enhancers can more easily enhance specific immune responses to protein antigens than to live viral vectors. This is because live viral vectors activate the natural immune response on their own. In our research, the unpolarized release of antigen was clear (Figs. 5 and 6) and the immune effect was more intense and widespread (Fig. 7). The increase in specific antibody responses were mainly due to the unpolarized release and not to the slightly nonspecific enhancement of immune responses.

In this study, anti-p24 gag antibodies served as a representatives of the mucosal humoral response. Although they cannot display traditional neutralization, they are very important to HIV vaccines. First, p24-specific IgA neutralizes HIV in cells intracellularly. This was proven in measles by one member of our team (unpublished data). She found that viral replication could be inhibited by binding of the internal protein M of measles by specific IgA in epithelial cells. This phenomenon was named intracellular neutralizing. Second, the p24 gag genes are relatively conservative, so their specific IgA is able to target more than one subtype of HIV. That of env cannot because its genes mutate readily.

This study explored the interactions between rVTT-gagpol and mucosal epithelial cell layers and their impact on antigen delivery and specific immune responses. Results demonstrate that rVTT-gagpol can infect epithelial cells to express p24 and that progeny virus and p24 are mainly released from the apical surface and both *in vitro* and in mice. The characteristic polarized distribution restricts the delivery of antigen and thus specific immune responses. To improve upon this, colchicine was use to depolarize distribution. This facilitated antigen expression, delivery, and specific immune responses. It has been reported before that polarized release of virus can restrict infection [45], but no research has yet been performed on the relationship between polarized release, antigen delivery, and specific immune responses.

These results provide an explanation for the low efficiency of rVTT-gagpol-induced mucosal immunity and supply some guidance for vaccine design with an eye toward increasing immune responses and decreasing dose. First, researchers may make use of colchicine (or other low-toxicity drugs) to increase viral and antigen distribution and in the epithelium and lamina propria. Second, they may develop recombinant vaccinia viruses using genes that target antigen budding from the basolateral surface, such as the M protein gene of VSV [46], [47]. Third, they may select viruses that are preferentially released from the basolateral surface, such as VSV, for use as vaccine vectors.
